# Spatial Release from Masking with a Moving Target

**DOI:** 10.3389/fpsyg.2017.02238

**Published:** 2017-12-20

**Authors:** M. Torben Pastore, William A. Yost

**Affiliations:** Department of Speech and Hearing Science, Arizona State University, Tempe, AZ, United States

**Keywords:** spatial hearing, sound localization, spatial release from masking, auditory motion, auditory salience, sound source localization

## Abstract

In the visual domain, a stationary object that is difficult to detect usually becomes far more salient if it moves while the objects around it do not. This “pop out” effect is important for parsing the visual world into figure/ground relationships that allow creatures to detect food, threats, etc. We tested for an auditory correlate to this visual effect by asking listeners to identify a single word, spoken by a female, embedded with two or four masking words spoken by males. Percentage correct scores were analyzed and compared between conditions where target and maskers were presented from the same position vs. when the target was presented from one position while maskers were presented from different positions. In some trials, the target word was moved across the speaker array using amplitude panning, while in other trials that target was played from a single, static position. Results showed a spatial release from masking for all conditions where the target and maskers were not located at the same position, but there was no statistically significant difference between identification performance when the target was moving vs. when it was stationary. These results suggest that, at least for short stimulus durations (0.75 s for the stimuli in this experiment), there is unlikely to be a “pop out” effect for moving target stimuli in the auditory domain as there is in the visual domain.

## 1. Introduction

In vision, the relative motion of objects provides a robust means to segregate foreground from background—e.g., Hillstrom and Yantis, 1994; Abrams and Christ, 2003. This paper investigates the extent to which the motion of a target sound source against a background of spatially separated, static sound sources might improve the recognition of the target compared to when the target does not move. That is, in a spatial release from masking paradigm, does target motion affect target recognition? This issue has been investigated for sentences by Allen et al. (2008) and Davis et al. (2016); the present study uses single words.

Allen et al. (2008) studied spatial release from masking using one target and two distractor Coordinate Response Measure (CRM) sentences (Moore, 1981; Bolia et al., 2000). These sentences begin with a call sign—one of eight names—which is then followed by a key word—the concatenation of one of four colors and one of eight numbers. In one of the tested conditions, the first portion of the masker sentences was played from loudspeakers at ±30°, while the target sentence was played from a loudspeaker directly in front of the listener, at 0°. The second halves of the masker sentences, however, were presented from the same 0° loudspeaker as the target sentence. Despite the co-location of the target and masker key words, the spatial separation of the call signs elicited a modest release from masking of 3.6 dB. Allen et al. (2008) concluded that the spatial separation between target and distractor for the initial, call sign portion of the target sentence enabled listeners to more effectively identify the characteristics of the target talker's voice and then maintain attention on that talker during the second half of the sentence when target and masker were co-located.

The movement of the words in the Allen et al. (2008) study occurred abruptly, between the first and last sets of words of the CRM sentence. Davis et al. (2016) investigated conditions in which either the target sentences or the distractor sentences moved while the other was presented from a stationary position. They used amplitude panning, based on the method of Pulkki and Karjalainen (2001), to rotate the moving target or distractor sentences around an azimuth array of loudspeakers. Several control experiments measured the extent to which motion, as opposed to just spatial separation, of target and masker sentences affected recognition of a target word. Davis et al. (2016) concluded that “in the presence of distracting messages, motion of either target or distracters and/or small spatial separation of the key words may be beneficial for sound source segregation and thus for improved speech recognition.” While Davis et al. (2016) used the appropriate controls, the magnitude of the effect of motion, *per se*, on speech recognition is still difficult to determine as the target and masker sounds were always at different locations for some period of time.

As Allen et al. (2008) point out, the explanation for their data is that listeners might gain information from the initial separation of target and maskers that could be used to facilitate streaming when they are not spatially separated. To some extent, this same basic issue could arise when interpreting the data of Davis, in that information at the beginning of the CRM sentences could aid in separating target from maskers at the end of the sentence. To minimize this possibility, we chose to present single words in a spatial release from masking paradigm, similar to the one employed by Yost (2017), in which listeners identified a female talker word in the presence of male masker words. The main independent variable in that study was the number of masker words (2, 4, or 6). Spatial release from masking decreased as the number of masker words (sources) increased. We wanted to determine if the effect, if any, of target motion depends on the number of spatially separated maskers. Therefore, two- and four-masker words were used in the present study.

This study reports a speech-on-speech masking experiment. Under such conditions, regardless of whether the target speaker and masking speakers are co-located or positioned separately, listener performance is usually assumed to be affected by a combination of informational and energetic masking—e.g. Kidd et al. (2008, 2016), Yost (2017) and, for diotically presented stimuli, Brungart et al. (2001). Energetic masking may be conceptualized as the reduction in audibility resulting from the spectro-temporal overlap of a target and its maskers. Informational masking has been rather broadly and variously defined—it may be simply thought of as masking that is not energetic masking, or as Watson (2005) put it, “…any threshold elevation not explained by overlapping patterns of neural excitation at or near the sensory receptors.” As such, informational masking is often thought of as the result of ‘higher level’ processing such as attention. For the purposes of the present discussion, informational masking occurs when a target and its maskers share features such as similar vocal characteristics and/or shared location, making it difficult for the listener to perceptually disentangle the target from its maskers. As a result, the listener's ability to attend to the target is compromised.

In the current study the female target word was moved using amplitude panning. The use of single words in the present study not only facilitates comparisions to Yost (2017), but short-duration words should either eliminate or greatly reduce the “conditioning” (i.e., streaming) information at the beginning of a sentence of many words—as used by Allen et al. (2008) and Davis et al. (2016)—in the hopes of getting closer to the effect of motion *per se*.

Identification of target words was measured at three target-to-masker level ratios chosen in pilot studies. An additional control experiment was conducted to determine if the direction of target word rotation could be discriminated in the presence of masker words.

When dynamically panning a target word, changes in masked thresholds may result from the motion of the target or the fact that the target is off-center for most of the presentation of the stimulus. To gain an estimate of the effect of presenting the target from a non-central location, a condition was included that presented the target statically panned to the furthest lateral extent of the dynamically-panned target condition.

## 2. Methods

### 2.1. Subjects

Fifteen listeners (10 females) between the ages of 19 and 37 (mean ≈ 23 years.) were tested. All listeners reported normal hearing. No listener had previously participated in any psychoacoustic experiment using the same testing paradigm. Participants were students in classes in the Speech and Hearing Science department at ASU (not taught by either of the authors). Students were offered extra credit in other, unrelated classes, if they chose to participate in the experiments. The procedures used in these experiments were approved by the Arizona State University Institutional Board for the Protection of Human Subjects (IRB).

### 2.2. Listening room

**Figure 1:**
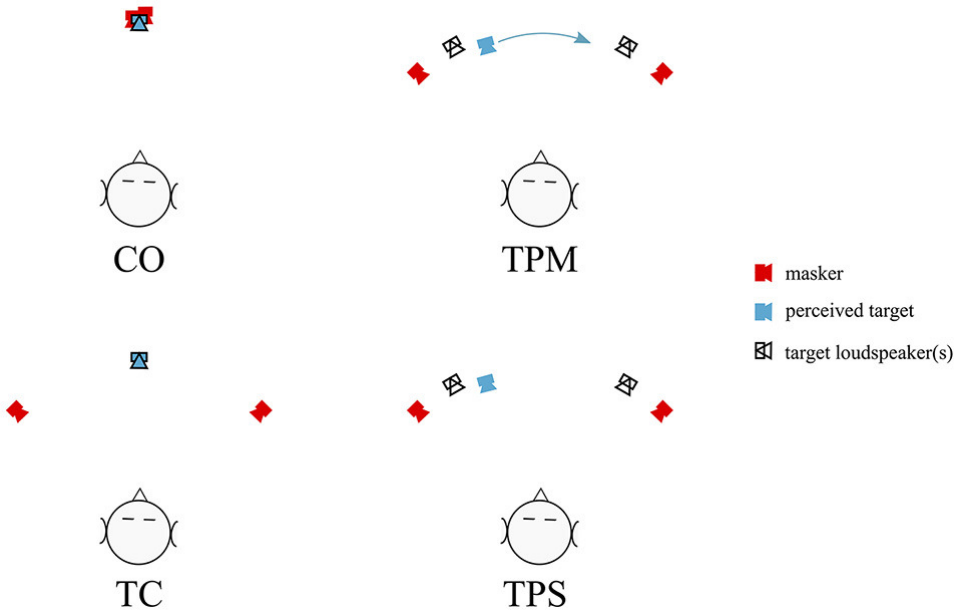
A schematic representation of the four basic stimulus conditions, shown for presentations with two maskers at ±45°. Presentations with four maskers added maskers at ±90°. **Upper left:** CO, the co-located condition, with target and maskers all presented from the same speaker at 0°. **Lower left:** TC, the target centered condition. Target is presented from the 0° loudspeaker, with maskers presented from side loudspeakers. **Upper right:** Target Panned Moving (TPM) condition. The target was amplitude panned using loudspeakers at ±30° to produce a “phantom” sound source moving between ≈ ±20°. **Lower right:** Target Panned Static (TPS) condition. Same as TPM, but the sound stimulus was statically panned to either ≈ ±20°.

The listening room was the same used for the SRM study of Yost (2017). It consists of 24 loudspeakers (Boston Acoustics 100X, Peabody, MA) on an azimuth circle 10 feet in diameter at pinna height (see Figure 1). The loudspeakers are located in a 15 × 12 × 10 foot room lined on all six surfaces with 4″ thick acoustic foam resulting in a room with a wideband reverberation time (RT_60_) of 102 ms. Listeners were seated in the middle of the azimuth array and were monitored via an intercom and camera from an adjoining control room. Listeners were asked to face the center loudspeaker, which had a red circle on it, and to keep their heads stationary. Listeners were closely monitored to be sure they did not move their heads and they rarely did so.

### 2.3. Stimuli

The same stimuli and procedure used in Yost (2017) were employed. Stimuli were 12 one word country names (Belgium, Britain, Burma, China, Cuba, Japan, Korea, Libya, Mexico, Norway, Russia, Turkey) spoken by one of six female or one of six male English speaking talkers. All words were bandpass filtered between 125 and 8,000 Hz (via a three pole Butterworth filter implemented in Matlab) and were centered within a 750-ms duration window, with silence filling those gaps that remained before and after the speech sample. The resulting 750-ms stimuli were all presented simultaneously for all conditions. All stimuli were initially normalized by their root-mean-square amplitude. All masker words were spoken by males and were presented so that the root-mean-square (rms) amplitude of the sum of all words measured at the center of room, where the listener sat, was approximately 65 dBA.

**Figure 2:**
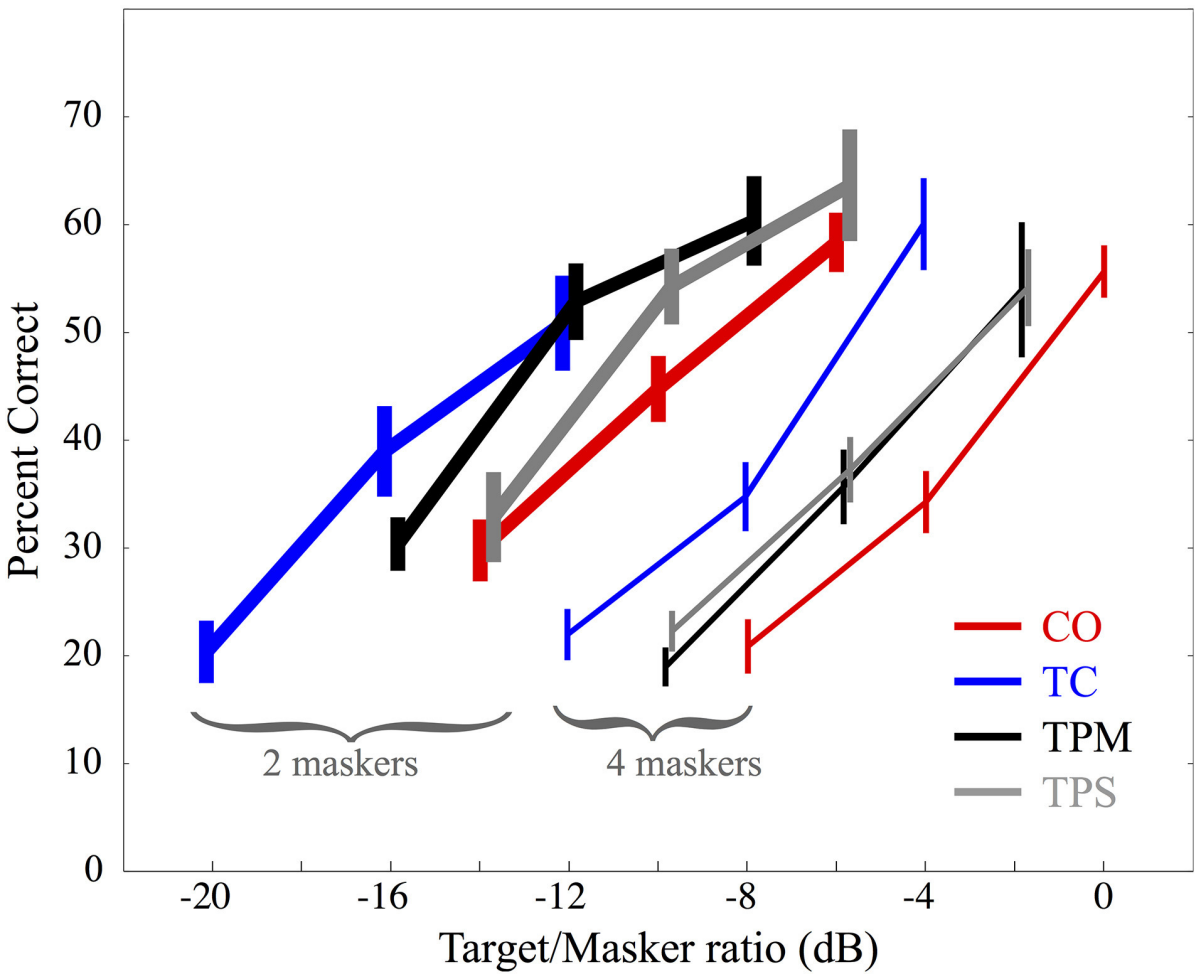
Mean data for 15 listeners performing a masked speech identification task with 2 and 4 maskers. Error bars represent ±1 standard error of the mean. For each condition, three target-to-masker decibel ratios, indicated along the horizontal axis, were tested. The four conditions are indicated by color: Co-located (CO) in red, Target Centered (TC) in blue, Target Panned Moving (TPM) in black, and Target Panned Stationary (TPS) in gray. Results for targets presented with 2 maskers are indicated by thick lines, whereas results for targets with four maskers are shown with thin lines. Error bars are staggered slightly to the left or right in order to facilitate legibility.

In Yost (2017) female target words were presented at 3 levels. In a pilot study, which presented a wide range of target-to-masker levels to three listeners, three testing levels, 4 dB apart from each other, were selected so that the proportion of words listeners correctly identified would be likely to lay between 0.2 and 0.8. This same method was used in the current study, using a new pilot study to arrive at levels appropriate to the conditions of the present study. The subjects who participated in the pilot testing were not used for testing in the main experiment, and the same stimulus parameters were used across all listeners. See Figure 2, for the tested target-to-masker level ratios.

The resulting data are three-point psychometric functions relating proportion correct to target-to-masker ratio expressed in dB. That is, target-to-masker ratio is the level of the target relative to the 65-dB SPL masker expressed in decibels. Female target words and male masker words were used to facilitate comparisons to Yost (2017) and, as in that study, to reduce the amount of informational masking—see also Brungart et al. (2001) for colocated target and masker and Kidd et al. (2016) for conditions where target and masker are positioned separately.

### 2.4. Set-up

As in the non-colocated target-masker conditions tested in Yost (2017), the maskers were placed symmetrically around the center target loudspeaker as shown in Figure 1. For the two-masker condition the maskers were at ±45° and for the four-masker condition at ±45° and ±90° (four maskers). In all conditions, masking words were chosen from the same 12 words used for the target, and the masker words were always different to each other and to the target word. Masking talkers were chosen from 6 different male talkers, so that, for each presentation, a random selection of two or four different talkers uttered the masking words. Target words were uttered by one of six different females, randomized between presentations.

Four conditions were run:

1. ***CO, Co-located condition***: All target and masker (two or four) words presented from the same center loudspeaker at 0°.
2. ***TC, Target Centered***: Target stationary at the center, 0°, loudspeaker. Maskers from loudspeakers at ±45° (two maskers) or ±45° and ±90° (four maskers).
3. ***TPM, Target Panned Moving***: Target moved from approximately ±20° to ∓20° (e.g., left to right or right to left) by amplitude panning using the loudspeakers at ±30°. Maskers from loudspeakers at ±45° (two maskers) or ±45° and ±90° (four maskers).
4. ***TPS, Target Panned Static***: Target statically panned to a single, stationary location of 20° to the left of center or 20° to the right of center using the loudspeakers at ±30°. Maskers from loudspeakers at ±45° (two maskers) or ±45° and ±90° (four maskers).

It was expected that there would be a release from masking when the targets were no longer located at the same loudspeaker as the target (CO vs. TC conditions), as reported commonly in the literature. This release from masking was then used as a reference by which to judge whatever release from masking might occur for the dynamically panned target condition (TPM). The statically-panned (TPS) condition sought to offer insight into what effect, if any, the off-center presentation of the target had in the TPM condition as compared with the TC condition. If motion of a sound source is like visual object motion then panning might affect target identification in these tasks.

Testing was blocked by condition, so that there were 8 = 4 *(conditions)* × 2 (*two or four maskers*) blocks of testing. For all conditions, stimuli were presented from left vs. right half the time, in random order. The same was true for dynamically panned stimuli: the movement was from left to right on half the trials, and vice-versa for the other half. The sine law of amplitude panning,—e.g., Bauer, 1961— was used with two loudspeakers at ±30°. The stimulus was presented simultaneously from both loudspeakers while the relative voltages at the two loudspeakers were adjusted to present a phantom sound source that moved between ±20°, with constant overall power. Grantham (1986) has shown that for low-frequency tones this panning method produces phantom sound sources with the same interaural differences as actual sources and listeners are unable to distinguish between phantom and actual sound sources.

### 2.5. Procedure

For each listener, all 72 possible combinations of target words and female target speakers (six female talkers by 12, one-word country names) were presented in random order for the three target levels used for each psychometric function. That is, as in Yost (2017), all 72 target words were used for each psychometric function so that different words were not sampled for different conditions. This results in 24 trials for each of the three points on the psychometric function. The masker words were also chosen randomly from the 72 possible masker words (six male talkers by 12, one-word country names). The words used for maskers or the target on any trial were always different and all 72 masker words (again six male talkers and 12, one-word country names) were sampled at least once for each psychometric function (see Yost, 2017). On each of the 24 trials, listeners used a keypad to indicate which of twelve words shown on a computer monitor was the word spoken by the female talker. Listeners could take whatever time they required to respond to any stimulus presentation. The next stimulus was presented 3 seconds after the listener indicated their response. Thus, for each listener there were 576 total trials [24 trials × 3 target-to-masker ratios × 4 masking conditions (CO, TC, TPM, and TPS) × 2 number-of-masker conditions (two and four maskers)]. The TPM and TPS conditions were blocked so that for half of the presentations sounds were presented starting on the right (TPM) or located as a stationary sound source on the right (TPS). The opposite occurred for the other half of the trials. Which side preceded the other was randomized, and the side the sound source was presented from was indicated on the computer monitor before each presentation to minimize any confusion on the part of the listener.

## 3. Results

### 3.1. Pilots

Several pilot scenarios were run to determine several of the parameters and conditions of this study. There were three experienced listeners (21–45 years old; one female) who participated in this pilot work with the author (M.T.P.) being one of them. The pilot work to determine target-to-masker ratios has already been described. All three listeners were immediately able, without practice, to discriminate the direction of movement of the panned target words when the target words were presented in isolation, with 100% accuracy. Data collected from other listeners, when the direction of panned movement was measured in the presence of masking words, is described in the Control Experiment section.

The pilot work also suggested that target word identification did not depend on whether the panned target words in the TPM condition moved left or right or whether the location of the stationary panned target word in the TPS condition was to the listener's left or right. Thus, the data in the main experiment were not analyzed in terms of the right-left randomization of target word movement (TPM conditions) or location (TPS condition).

### 3.2. Main results

Figure 2 shows the three-point psychometric functions displaying mean and ± one standard error of the mean for the proportion of correct target words identified as a function of the three target-to-masker ratios (dB) for 15 listeners. Thick lines indicate results for the two masker conditions and thin lines show results for the four masker condition. The Collocated (CO) condition is shown in blue, the Target Centered (TC) condition is shown in red, the Target Panned Moving (TPM) condition is shown in black, and the Target Panned Stationary (TPS) condition is represented in gray.

For both the two and four masker conditions, there is less masking when target and maskers are presented from loudspeakers at different locations. This spatial release from masking (SRM) is evidenced by the leftward shift of the psychometric functions for TC as compared with CO.

For the two-masker condition, dynamic panning for the TPM condition offers no decrease in masking; listener performance is quite similar to the TC condition. There was somewhat less of a release from masking when the target was statically panned in the TPS condition. However, the relatively high variability in listener response, and the fact that there was no systematic trend across individual listeners' data for the TC, TPM, and TPS conditions suggests that target word motion using panning may not lead to any reliable increase or decrease in masking beyond that which is obtained by spatially separating a target from the maskers.

For the four-masker condition, outcomes were quite similar to the two-masker condition, except that identification thresholds were all shifted to higher target-to-masker levels. That is, Consistent with Yost (2017), there is clearly more masking when the target is presented with four rather than two maskers. With four maskers, there was practically no difference in performance between the statically panned and dynamically panned target conditions, further suggesting no effect of target movement *per se* on identification accuracy.

As in Yost (2017), the psychometric functions in Figure 2 appear somewhat linear. Furthermore, all psychometric functions show basic trends similar to those found in Yost (2017). That is, in the current study the mean slope across all conditions and listeners was slightly less than in Yost (2017) (0.041 vs. 0.048 proportion correct/dB) and the standard deviation was slightly higher (0.040 vs. 0.028 proportion correct/dB).

A within-subjects analysis of variance (ANOVA) of the slopes of the mean, across-subjects linear regressions for each of the four conditions for both number-of-masker conditions indicated that there was no statistically significant difference (*p* = 0.061, α = 0.05). The correlation coefficients for the linear regressions ranged from 0.915 to 1.0 indicating that the psychometric functions were linear or nearly so. Thus, straight-line fits of proportion correct vs. dB were used to fit all psychometric functions.

Spatial release from masking (SRM) was calculated for each condition by finding the target-to-masker ratio required for 0.54 proportion correct (the midpoint between chance, 1/12 = 0.0833, and perfect performance) for the CO condition and subtracting this value from the same measure of performance in the condition of interest. SRM measured in this way for symmetrical maskers was quite similar for the four-masker TC condition (4.19 dB) and the two-masker TC condition (4.77 dB). The moving the target (TPM), yielded an SRM of 1.86 and 3.06 dB for the two and four masker conditions, respectively. Panning the target off-center, but stationary (the TPS condition) elicited little change in performance, at 1.91 and 1.73 dB for the two and four masker conditions.

**Figure 3:**
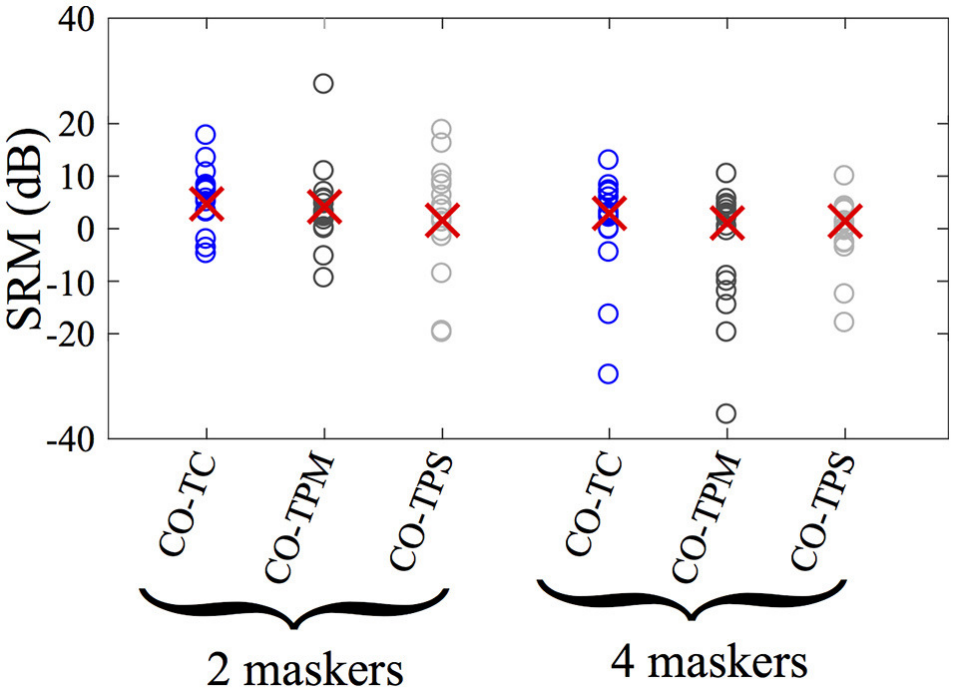
Spatial release from masking (SRM) for 15 listeners. Circles represent individual listeners' performance and red x's represent the group mean. SRM is calculated as the difference between the target-to-masker ratios required for the 54% correct for the CO condition and the experimental conditions referenced in the horizontal axis label. See the Results section for further details of this calculation.

Figure 3 shows these mean values as well as individual results for SRM for all 15 listeners for the TC, TPM, and TPS conditions in the presence of two and four masking talkers. While the mean spatial release from masking is greatest for the two-masker condition with the target in the center, the variability between subjects within each condition is far greater than the difference between mean values in any comparison across conditions. Note also that mean SRM is less for both panned target conditions (TPM and TPS) than their respective TC conditions, for both the two and four masker cases, although again, variability is far greater than these differences. Looking at individual listeners across conditions (not shown) revealed no clear trend. That is, some listeners demonstrated increased SRM with panning of the target and others demonstrated reduced SRM with panning of the target.

Statistical analyses of the two- and four-masker pooled individual data were performed separately. The target-to-masker ratio required for a 0.54 proportion correct was obtained for each of the three point psychometric functions based on a linear best fit to each listener's functions. These target-to-masker ratios were subjected to a one-way (four target conditions of CO, TC, TPM, and TPS), repeated measures ANOVA—one for the two-masker data and one for the four-masker data. There was a statistically significant difference due to target condition for both number of masker cases (*p* = 0.018 for the two-masker case, *p* = 0.043 for the four-masker case). Paired-wise, post-hoc t-tests with Bonferroni correction were computed comparing all 6 possible pairings of the 4 (CO TC TPM TPS) conditions for each number of masker cases (two and four maskers). Only the difference between the CO and TC conditions for each number of masker cases reached statistical significance. This reinforces the observation from Figure 2 that the difference between the CO and TC condition was significant, suggesting that there was significant SRM, but that neither of the panning conditions produced a significant difference from either the TC (spatially separated masker only condition) or the CO condition. Analyzed in terms of SRM, there was also no significant difference, even with no correction for family-wise error, between the three SRM conditions. All 15 of the 15 listeners in the 2 masker conditions and 12 out of 15 in the 4 masker conditions had target to masker ratios that were less for the TC than the CO condition. No similar trends existed for any of the other pairwise comparisons.

## 4. Discussion

This study tested listeners' accuracy in identifying a single target word, spoken by a female, presented with two or four symmetrically spaced masker words spoken by males under conditions where the target was panned from one side to the other (TPM condition) or was presented from the center 0° loudspeaker (TC condition). The main finding of this study was that there was no statistically significant difference between these two conditions, both in terms of percent correct and SRM. That is, we found no evidence that target motion enhances target identification for the short speech stimuli presented in this study, regardless of whether there are two or four maskers. This result is in opposition to what might have been expected based on the phenomenon of visual “pop-out.” This is also in contrast to the interaction with masker number that exists for static targets, as shown in Yost (2017). The control TPS condition did not produce identification performance that was statistically any different to that provided by either the TC or TPM condition, again suggesting that spatial separation of the target from the maskers, not target movement *per se*, was the main driver of listener performance.

Consistent with Yost (2017), SRM between the CO and TC was statistically significant for both the two- and four-masker conditions, and SRM was roughly the same whether there were two or four maskers. Listeners demonstrated a slightly reduced SRM with poorer, more variable overall performance than did the listeners in Yost (2017). These small differences are likely to be the simple result of using a different set of listeners in the two studies, as the studies were conducted in different years.

It is possible that the panned motion of the target was simply not sufficient enough in one way or another (e.g., fast enough, long enough, over a long enough arc, etc.) to induce a change in word identification. The target word moved 40° at a rate of 53.33°/s over a 0.75-s period. These parameters are well within the limits of those used to measure the minimum audible movement angle (MAMA)—e.g., Grantham, 1986; Perrott and Tucker, 1988; Chandler and Grantham, 1992—suggesting that the motion should have been detected, as confirmed by the pilot study. However, such target word motion may not be as easily detectable when there are masker words also present. To confirm that listeners could in fact perceive the motion of the target for the panning conditions with a masker present, a control experiment was performed.

## 5. Control experiment

The listeners in the two-masker TPM condition were asked to discriminate whether the panned target word moved clockwise (rightward) or counterclockwise (leftward). Six listeners who reported normal hearing (ages 19–45, two females, median age 28 years) were tested. The same words were used as were used in the main experiment. Also, the same set up was used as was used in the main experiment.

The two-masker TPM condition was tested in which listeners indicated if the panned target word moved left (counter-clockwise) or right (clockwise). Half of the 72 trials moved leftward and the other half rightward, and they were presented in random order. The target word was panned as in the TPM condition in main experiment, but the level of the target word relative to the masker words was +2 dB, 8-dB greater than it was for the largest target-to-masker ratio used in the main experiment (for the CO condition, see Figure 2).

Percent correct indication of leftward or rightward target motion ranged from 69% to 100% with a mean of 84% across the six listeners. Thus, an inability to discriminate target motion does not seem to explain the lack of an appreciable effect of target motion on identification performance for the SRM context tested in this study.

The results of the current study are not consistent with those from the Davis et al. (2016) study, in which moving sentences *did* produce a small identification advantage over not moving the sentences. However, there are many differences between the two studies (e.g., words vs. sentences, when spatial changes were made, rate of motion, whether target and/or maskers rotated, etc.), and any one or some combination of these differences could account for the divergent results. One such difference is the duration of the target, seconds-long sentences in the Davis et al. (2016) study and less than a second in the current study. It might take a relatively long time to effectively process auditory motion so that the motion assists identification, and the 0.75-s time period used in the current study may be less than listeners require to benefit from motion of the target source.

## 6. Overall discussion

In the visual literature, the motion of a visual target stimulus embedded with other visual masking stimuli has been shown to increase the salience of the target stimulus so that it “pops out.” This study sought to determine if there might be any analogous effect for auditory stimuli. We found no evidence to suggest that this is the case for short-duration word sounds. As discussed above, while the duration of the sounds is short and the motion covers only 40°, this motion is detectable and is consistent with that used in other studies of auditory motion—e.g., Grantham (1986). However, since there are very few studies of sound identification of moving sound sources it is probably premature to generalize beyond the findings of this investigation.

There is a literature—e.g., Grantham and Wightman (1979)—suggesting that binaural processing is a “sluggish” process, that fast changes in auditory spatial cues are not processed as moving sounds or sound images. Most studies of “binaural sluggishness” have used stimuli delivered via headphones. In a sound field such as the one used in the current study, Yost and Brown (2013) used two broadband noises at different locations that were sinusoidally amplitude modulated out-of-phase such that when the noise at one location had maximal intensity the noise at the other location had minimal intensity. Listeners' ability to localize the two out-of-phase modulated noise sources was more accurate than when the two noises were modulated in phase at rates as high as 200–500 Hz. However, listeners were unable to determine which noise source produced the louder sound (i.e., determine the direction of movement between the two sources) for modulation rates greater than 25–50 Hz. That is, the ability to determine the actual motion between the two sources was limited to rates of motion less than 50 Hz. This finding is consistent with the “binaural sluggishness” literature (Yost and Brown, 2013). Perhaps “binaural sluggishness” limits the ability of the auditory system to process sound source motion unless the motion is extremely slow–as such the 53.33°/s rate of motion used in this experiment may be too fast.

Unlike the study of Davis et al. (2016), target and masker in the present experiment were of opposite sexes, presumably leading to a reduction in informational masking—e.g., Brungart et al., 2001 and Kidd et al., 2016. It may be that the assumed reduction in informational masking for the present study left little room for the movement of target words to induce any additional improvement in listener performance. Nevertheless, the precise degree to which energetic vs. informational masking is reduced by using a target talker of the opposite sex from the maskers is unclear, and so speculation should be approached cautiously.

A direct comparison of the results of this study with those of Davis et al. (2016) is highly problematic. For one thing, Davis et al. (2016) presented all target and masker stimuli at the same level (e.g., 0-dB signal-to-noise ratio), whereas stimuli were presented at three different levels for each condition in the present study. Also, Davis et al. (2016) located two maskers either at 0°, directly in front of the listener, or 60° to the left of the listener. The target stimulus then moved either toward or away from the maskers. By contrast, in the present study, maskers were symmetrically placed to the left and right of the listener and the target moved *between* the maskers, so that as the target moved away from one masker, it moved toward the other. It may be that the benefit from movement found by Davis et al. (2016) works under acoustic scenarios where a better-ear advantage can be used to segregate target from maskers, whereas the symmetrical placement of maskers in the present study largely removed any better-ear cues a listener might use. In Davis et al. (2016), there is no simple condition where target and maskers are all stationary but placed at different locations, so it is difficult to evaluate how performance would have been affected by spatial separation without target motion.

Comparisons with Allen et al. (2008) are somewhat more straightforward, but again there are considerable differences in experimental design between the present report and that of Allen et al. (2008). First, while Allen also placed maskers symmetrically to the left and right of the listener (for a total of two maskers), the maskers moved while the target remained stationary. As such, both maskers either moved toward or away from the target. As in Davis et al. (2016), CRM sentences were presented, so that the target stimulus could be located separately from the maskers during the initial words of the sentence but co-located with the maskers during presentation of the keywords listeners needed to identify, or the opposite case. When the target was stationary and located at the center, with stationary maskers to the left and right of the listener at ±30°, speech reception thresholds (SRT) were reduced by an average of 12 dB as compared with the condition where target and maskers were stationary and co-located. Similarly, for the condition where target and maskers started at the same location and then the maskers were moved away from the target for presentation of the keywords listeners were asked to identify, an average unmasking of 11 dB was reported. When instead the first part of the CRM sentence was presented with maskers separated from the target and the maskers then moved into a co-located position with the target for presentation of the key words, the reduction in SRT was 3.6 dB, on average. The clearest outcome of the Allen et al. (2008) study is that separation of the target and maskers at the time of presentation of the keywords conferred the greatest benefit to listeners. It is especially noteworthy that there was no statistical difference in listener performance between the condition where target and maskers were separated for the entire duration of stimulus presentation and the condition where target and maskers started co-located but separated before presentation of the keywords. The results of the present study, which showed a benefit to spatial separation of target and masker but no strong evidence for any benefit from target motion *per se*, are in this way consistent with those of Allen et al. (2008).

As mentioned in the introduction, the stimuli presented in Allen et al. (2008) and Davis et al. (2016) were full sentences, exceeding the duration of the single-word stimuli presented in the current study several-fold. These longer-duration stimuli may have allowed listeners the time to form “auditory streams” that would have lead to a greater release from informational masking than was allowed for the single-word stimuli reported in this study.

Kidd et al. (2016) found that spatial separation of two or four speech maskers and a speech target lead to a reduction in masking as compared to conditions where maskers and target were co-located. This masking difference was estimated to be largely the result of a reduction in informational masking in that spatial separation allowed listeners to perceptually group target and maskers into separate “streams” of information—see also Arbogast et al. (2002). The study of Davis et al. (2016) tried to pick apart the relative contributions of target movement vs. placing target and maskers at different locations. Results showed that the mere separation of target and maskers, with or without movement, was likely to account for much of any release from masking. The results of the present study add support to this view. That is, movement of the target appears to offer no further reduction in informational masking beyond that which is already provided by the spatial separation of target and maskers. Regardless, these speculations should be considered most cautiously, preferably as motivation for new, targeted data. Future experiments could test whether similar results would occur in a masking context that was more energetic (e.g., using noise maskers) or more informational (e.g., using the same talker, or same-sex talker for targets and words). Word identification was measured in the current study, and it is not clear if similar results would occur if a detection or a discrimination task was used.

Taken together, the results of this study, the results of Davis et al. (2016) study indicating a small effect of sentence motion on speech identification, and the “binaural sluggishness” literature do not provide strong evidence to suggest that movement of a sound source in a sound field, at least over short durations, is a powerful cue for segregating one sound source from other sources. This is in contrast to the visual literature in which relative motion of visual objects is a powerful means of segregating visual objects. This may have its root in the difference between the spatiotopic encoding at the retina vs. the nature of auditory space, which requires the computation of sound source location using binaural difference cues and spectral cues in combination with other systems/sensory inputs to register the location of the listener's head in space. Nevertheless, there are so few studies of sound source motion in a sound field that it is probably premature to make strong claims about the role of sound source motion in auditory perception.

## Author contributions

This article was co-designed, programmed, analyzed and written by WY and MP using the experimental approach of Yost (2017) as a guide.

### Conflict of interest statement

The authors declare that the research was conducted in the absence of any commercial or financial relationships that could be construed as a potential conflict of interest.
